# Addressing the Leadership Gap: A Systematic Review of Asian American Underrepresentation in Orthopaedic Surgery

**DOI:** 10.3390/healthcare13161987

**Published:** 2025-08-13

**Authors:** Ahmed Nadeem-Tariq, Matthew Michelberger, Christopher J. Fang, Jeffrey Lucas Hii, Sukanta Maitra, Brock T. Wentz

**Affiliations:** 1 Kirk Kerkorian School of Medicine at University of Nevada Las Vegas, 625 Shadow Lane, Las Vegas, NV 89106, USA; christopher.fang@unlv.edu (C.J.F.); jeffreylucas.hii@unlv.edu (J.L.H.); brock.wentz@unlv.edu (B.T.W.); 2 College of Sciences, University of Nevada Las Vegas, 4505 South Maryland Parkway, Las Vegas, NV 89154, USA; matthew.michelberger26@gmail.com

**Keywords:** Asian American, AAPI, orthopaedic surgery, workforce disparities, professional development

## Abstract

**Background**: While Asian American individuals are well represented in medical schools in the United States, their advancement to senior positions within the field of orthopaedic surgery is disproportionately low. This underrepresentation not only limits diversity in leadership but also constrains the development of people-centred systems that reflect the needs of an increasingly diverse patient population. **Objectives**: This study systematically examines Asian American representation across the orthopaedic surgery professional pipeline, focusing on disparities between training-level representation and advancement into both faculty and leadership positions., and framing these gaps as a health equity concern. **Methods**: A comprehensive literature search for peer-reviewed original research articles was conducted via PubMed, EBSCO Open Research, Wiley Online Library, Google Scholar, and ScienceDirect. The potential articles were screened against prespecified eligibility criteria, and risk of bias was assessed using the Newcastle–Ottawa Scale (NOS). Data were then systematically extracted and analysed. **Results**: This analysis included 20 research articles investigating Asian American representation in orthopaedic surgery. The results demonstrated an underrepresentation of Asian Americans in orthopaedic leadership positions despite improvements in training programme representation with subspecialty clustering in adult reconstruction and spine. Asian American surgeons were less likely to occupy academic and leadership roles than their non-Asian American peers. Across studies, underrepresentation was consistently observed, with effect size estimates indicating a substantial disparity (e.g., pooled risk difference = 0.19; 95% CI [0.12, 0.28]) in those studies reporting comparative outcomes. Similarly, while Asian Americans in residency programmes increased, this growth did not translate proportionally to faculty advancement. In contrast, Asian women face compounded barriers, particularly in subspecialties like spine surgery. These inequities undermine workforce inclusivity and may reduce cultural and linguistic concordance with patients. **Conclusions**: Despite having strong representation in orthopaedic training programmes, Asian Americans are disproportionately absent from leadership positions. This poses a challenge to equity in surgical education and patient-centred care. To promote equity in leadership, focused mentorship, clear promotion processes, and institutional reform are necessary to address structural barriers to career advancement, this will reflect the diversity of both the workforce and populations served.

## 1. Introduction

The U.S. healthcare workforce continues to fall short in reflecting the country’s growing ethnic diversity [1]. Adequate representation of racial and ethnic minorities in medicine is important for improving physician–patient communication, increasing patient satisfaction, and promoting adherence to treatment plans through cultural concordance [2,3] but also for advancing health equity and building people-centred systems that deliver care responsive to the needs of diverse populations. In recent years, there has been a growing acknowledgment of the importance of physician diversity in addressing healthcare disparities, fostering cultural competence, and encouraging innovation [4,5,6]. However, significant gaps in representation persist across medical specialties.

Orthopaedic surgery remains one of the least diverse fields in medicine, with consistently low representation of both women and racial or ethnic minorities [7]. Contributing factors include structural barriers such as limited exposure to the specialty during medical training and ongoing concerns about work–life balance and workplace culture [8,9]. These barriers have downstream effects on the inclusivity of surgical leadership and by extension, the capacity of orthopaedic institutions to implement policies that prioritise equitable patient-centred care.

Asian American communities comprise a complex and heterogeneous group in medicine. Although they represent approximately 20% of U.S. medical school graduates, Asian Americans constitute only about 7% of the general population. There is an apparent overrepresentation in entry-level roles which conceals disparities in leadership and advancement [10,11]. The “model minority” stereotype often ascribes technical competence while downplaying leadership potential, reinforcing biases that limit access to senior and decision-making roles [12,13].

In orthopaedic surgery, Asian American physicians face distinct patterns of exclusion that differ from those experienced by other underrepresented groups [14]. While they may gain initial access to training programmes, systemic barriers often impede their advancement into leadership positions [15]. Persistent stereotypes that portray Asian American surgeons as technically proficient but lacking leadership qualities may contribute to their exclusion from roles of influence and recognition, limiting opportunities for career growth [16].

Despite increasing awareness of diversity challenges within orthopaedics, there is a lack of focused research on the professional trajectories and leadership representation of Asian American surgeons. While previous studies have outlined general demographic trends, few have examined the progression of Asian American physicians across the orthopaedic pipeline—from medical training through to leadership roles along with the implications of these disparities for equitable, and people-centred healthcare delivery.

For the purposes of this review, leadership refers to roles such as professorships, fellowship directorships, and elected positions within national orthopaedic organisations. This systematic review aims to fill that gap by evaluating the representation of Asian American individuals in orthopaedic surgery, with a focus on their career progression and the systemic factors influencing their access to leadership.

One conceptual lens through which these disparities can be examined is the “bamboo ceiling,” a term coined to describe the invisible barriers that impede career advancement for Asian professionals, particularly in leadership roles, despite strong educational or technical qualifications. In academic medicine and surgery, this phenomenon may manifest through institutional biases that stereotype Asian physicians as competent but lacking the interpersonal or assertive traits perceived as prerequisites for leadership. Incorporating this framework allows for a more nuanced interpretation of career progression and structural exclusion in orthopaedic surgery, particularly for Asian American individuals, and situates the findings within a broader health equity and people-centred systems context.

### 1.1. Research Objectives

To evaluate the representation, career advancement patterns, and systemic barriers facing Asian American individuals across the orthopaedic surgery professional pipeline, from residency training through academic leadership positions.

### 1.2. Research Question

- What are the trends and disparities in Asian American representation in orthopaedic surgery, academic, and leadership positions?
- How do intersectional factors of race and gender impact career advancement opportunities and leadership attainment for Asian Americans in orthopaedic surgery?

## 2. Methodology

This study adhered to the Preferred Reporting Items for Systematic Reviews and Meta-Analysis (PRISMA) [17]. A review protocol was entered into the OSF database (registration DOI: https://doi.org/10.17605/OSF.IO/2ZT84; date registered: 21 July 2025).

### 2.1. Identification and Selection of Studies

We searched PubMed (MEDLINE), CINAHL via EBSCOhost, Wiley Online Library (journals platform), and ScienceDirect between 15 January 2025 and 1 February 2025, for literature published January 2000–January 2025. A supplementary search of Google Scholar was conducted using the query: ‘Asian American orthopaedic surgery representation leadership’ The first 200 results (equivalent to the first 20 pages) were screened manually for relevance. Inclusion was limited to English-language peer-reviewed research articles published between 2000 and 2025, with studies selected if their titles or abstracts mentioned AAPI representation, diversity, or leadership within orthopaedic surgery.

### 2.2. Search Strategy

A pilot study was conducted through Google Search and academic journals to identify keywords related to Asian Americans, Asian American physicians, and orthopaedic surgery. The identified keywords were combined using Boolean operators and used with MeSH terms to formulate search strings. Search strings were pilot tested for their reliability to retrieve relevant articles on the representation of Asian Americans in orthopaedic surgery.

### 2.3. Study Selection

The retrieved results were exported to Zotero screening software version 6.0.36. The software automatically detected, retracted articles and compared metadata to identify duplicate records, which were manually merged. Two reviewers then independently selected the non-duplicate records through a two-step screening and selection process: title and abstract screening followed by full text screening against prespecified eligibility criteria. Conflicts in the selection process were resolved through discussion or the intervention of a third reviewer whenever necessary.

### 2.4. Eligibility Criteria

#### 2.4.1. Inclusion Criteria

This study included research on the representation, career advancement patterns, and leadership attainment of Asian American individuals in orthopaedic surgery. Articles fulfilling the modified PICOS criteria were selected [18].

The PICOS criteria for eligible studies were defined as follows:

Population (P): Asian American orthopaedic surgeons, residents, fellows, faculty members, patients, orthopaedic leadership bodies, and academic institutions.

Intervention (I): No intervention.

Comparison (C): Representation, advancement patterns, and leadership attainment comparing Asian Americans to other racial/ethnic groups in orthopaedic surgery.

Primary outcomes (O): Proportion of Asian Americans in training programmes, faculty positions, leadership roles, progression rates, subspecialty distribution, and barriers to advancement. For the purpose of this review, “leadership” was operationally defined as attainment of roles with institutional decision-making authority or influence within the field of orthopaedic surgery. This included academic ranks of associate or full professorship, positions on boards of directors or editorial boards of orthopaedic societies or journals, fellowship directorships, and elected leadership within national subspecialty organisations (e.g., AAHKS, Hip Society, POSNA). These roles were selected as valid indicators of leadership based on their influence over research agendas, trainee mentorship, policy setting, and representation within the profession.

Study Design (S): Retrospective analyses, cross-sectional studies, longitudinal cohort studies, and survey-based research examining racial representation in orthopaedic surgery.

#### 2.4.2. Exclusion Criteria

This study excluded non-research articles such as letters, editorials, conference abstracts, and opinion pieces; studies not available in English; research that did not specifically report data on Asian American representation in orthopaedic surgery; study protocols, narrative reviews, and non-systematic literature reviews; and research exclusively examining non-orthopaedic surgical specialties.

### 2.5. Methodological Quality Assessment

We used the Newcastle–Ottawa Scale (NOS) to appraise observational studies. For cross-sectional roster analyses that do not fit NOS domains perfectly, NOS items on representativeness and outcome ascertainment were applied, and we report these appraisals descriptively. We acknowledge that design-specific tools (e.g., AXIS for cross-sectional studies) could provide additional nuance and note this as a limitation.

### 2.6. Data Selection and Extraction

A two-reviewer system was used to systematically extract and organise data from the included studies using Microsoft Excel 2024. The first reviewer extracted the data while the second reviewer verified the data for consistency and accuracy. The key variables were collected, including the study ID, study design, sample size, demographic breakdown, study objective, and conclusions. This structured extraction process ensured consistency across studies and facilitated comprehensive analysis and comparison of the included literature.

### 2.7. Data Analysis

Extracted data were analysed using a structured thematic synthesis approach. The first reviewer organised findings across included studies into predefined domains relevant to the review objectives namely, Asian American representation in training programmes, academic advancement, leadership roles, and intersectional gender-based disparities. These thematic categories were refined through iterative discussion with co-authors to ensure internal consistency and conceptual clarity.

While formal qualitative coding techniques (e.g., line-by-line annotation or software-assisted coding) were not employed, this thematic grouping allowed for coherent synthesis of key trends across studies with heterogeneous designs and outcome measures. Additionally, quantitative frequencies of representation trends were descriptively summarised when available to support the thematic analysis. Due to substantial heterogeneity across study populations and outcome definitions, meta-analysis was not pursued [19,20]. While an initial pooled analysis of underrepresentation was conducted, substantial heterogeneity in study design, leadership definitions, and outcome measures (I^2^ = 98%) limited the interpretability of the pooled estimate. As such, we opted to present a narrative synthesis of findings to better capture the complex and context-specific nature of Asian American disparities in orthopaedic surgery.

## 3. Results

### 3.1. Study Selection

**Figure 1:**
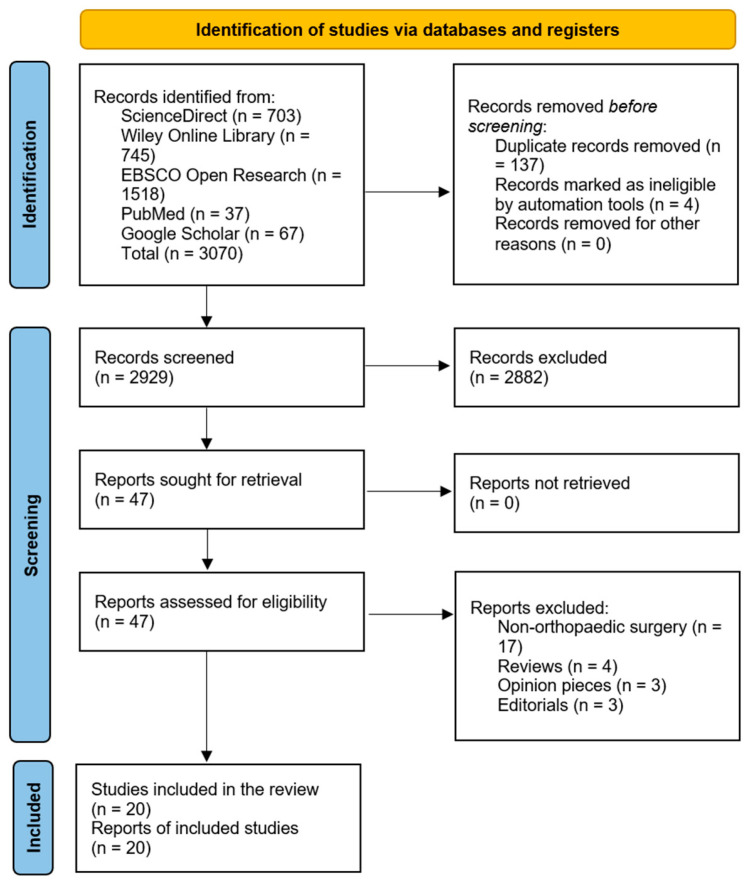
PRISMA flow diagram [1].

The database search retrieved 3070 records. Non-duplicate records were screened, after which 2882 records were excluded. Forty-seven full-text articles were screened, after which 20 research articles were included. Full-text exclusions (*n* = 27) comprise the categories shown in the right-hand box. Studies included in the review (*n* = 20) are displayed in the lower box. The total full text assessed for eligibility = 47 (Figure 1).

### 3.2. Methodological Quality Assessment

Overall, the methodological quality of the 20 included studies was moderate to high. The majority of studies scored between 6 and 8 out of 9 on the Newcastle–Ottawa Scale (NOS), indicating low to moderate risk of bias. All studies adequately addressed cohort representativeness and outcome ascertainment, although comparability across cohorts was inconsistently reported, particularly regarding adjustments for confounding variables. No studies were excluded on the basis of NOS score. However, studies with higher NOS scores were given greater interpretive weight in our synthesis of leadership trends.

While all 20 studies were included in the final review, only 16 directly evaluated leadership-related outcomes such as academic appointments, executive roles, or program leadership. The remaining four studies, though relevant to broader AAPI representation, focused on clinical utilization patterns or procedural outcomes and were excluded from the thematic synthesis to preserve conceptual clarity (Table 1).The studies included in the thematic analysis and summary are listed in Table 2. 

### 3.3. Data Selection and Extraction

The present study included 20 retrospective analyses published between 2010 and 2025 examining Asian American representation in orthopaedic surgery. The studies covered multiple domains: residency programs, fellowship training, academic faculty, leadership positions, and patient care. Most studies compared demographic representation against population benchmarks, while others examined career advancement metrics (Table 2). This table helps to break down barriers towards leadership presented in various studies and lays out demographic comparisons in percentage form.

### 3.4. Thematic Analysis of Outcomes

Underrepresentation in Academic and Leadership Roles.

Asian American surgeons remain significantly underrepresented in academic and leadership positions within orthopaedic surgery, despite their presence in the field. Among 186 academic shoulder and elbow surgeons, Asian professionals only composed 14.5% of the participants, while 83.9% were White [20]. This underrepresentation was more pronounced at senior academia ranks, where 21.7% of the Asian surgeons are professors compared to 44.0% of White surgeons (*p* = 0.04) [20]. Analysis of 94 adult reconstruction fellowship directors (FD) revealed that 99% of the leadership were males, with only one woman in a leadership role. As far as racial representation goes, 80.65% were Caucasian, 12.90%.

Asian American, 5.38% Hispanic/Latino, and 1.08% African American [30]. Similarly, among presidents elected to the Hip Society, Knee Society, and AAHKS between 1990 and 2022, 95% were Caucasian, with only 2% Asian and 3% Hispanic representation [32]. At the same time, Asian Americans were 12.0% of registered orthopaedic residents overall, but they only made up 9.1% of the faculty, suggesting attrition in academic progression [22].

Asian American minorities were also missing in orthopaedic surgery practice beyond academia. Relative to other medical specialties, fewer Asian individuals were training in orthopaedic surgery, at only 12.0% of residents, indicating exclusion at the entry and mid-career points [23]. There are notable experiences of discrimination, as 20.7% of orthopaedic trainees report racial or ethnic bias during training. These experiences were reported more frequently by trainees who are minorities, which further shows ongoing challenges related to inclusivity and equity in the field [22].

Across the 18 included studies, a consistent trend was observed: Asian American individuals were well represented in orthopaedic training programmes but significantly underrepresented in senior academic and leadership roles. These disparities were especially notable in subspecialty societies, fellowship director roles, and among NIH-funded researchers. Several studies explicitly reported statistically significant gaps in promotion or tenure for Asian American surgeons, even when controlling for years in practice or publication volume.

### 3.5. Representation Trends over Time

Representation in the Training Pipeline: Medical Students and Residents.

Asian American surgeons are consistently well represented among medical students in the United States, accounting for over 20% of medical school graduates, although making up just about 7% of the population. This high level of representation indicates a good entry into the medical education pipeline, demonstrating that initial access is not the primary barrier to specialty selection or advancement. However, this representation falls dramatically in orthopaedic surgery, indicating that inequities emerge early in the specialty-specific training track.

Asian American representation in orthopaedic residency programs has increased gradually over time. Between 1995 and 2008, this group increased from 7.24% to 13.14%, indicating a moderate incremental gain of 4.53% each decade [27]. Despite this increase, orthopaedic surgery has regularly ranked as one of the least diverse specialties. For example, in 2008, underrepresented minorities (URMs) accounted for 20.2% of all medical residents, but Asian Americans accounted for only 11.7% of orthopaedic trainees [26].

From 2007 to 2019, the percentage of Asian Americans applicants for orthopaedic surgery increased from 11.7% to 14.7%. This development, however, did not result in proportional gains among matched applicants, whose numbers increased by only 3% throughout the same period [27]. The disparity between applicant volume and successful matriculation raises questions about potential impediments to the selection or review process.

Similarly, from 2006 to 2015, Asian American representation among orthopaedic residents stayed steady at 12.5%, with no significant rise over a decade, whereas other surgical specialties experienced more consistent diversity gains [29]. Further longitudinal data from 2001 to 2020 demonstrate that Asian Americans representation among newly matched orthopaedic residents ranged between 10.4% and 15.4%, with no continuous increasing trend during the 19 years [35].

There is a stark contrast between this stagnation and the continuous increase in Asian Americans representation in medical schools, which may imply that orthopaedic surgery serves as a bottleneck in the overall training pipeline. While there may be many causes of this stagnation, the data show that access to orthopaedic residency does not mirror the strength of the Asian American medical student population. Limited early exposure, perceived cultural fit, mentorship discrepancies, and implicit bias may all contribute to this mismatch.

### 3.6. Attending and Leadership Representation

Asian Americans make up a considerable proportion of orthopaedic residents, but their presence decreases significantly at the teaching and leadership levels, demonstrating a clear attrition trend as career stages progress. Despite joining orthopaedics at comparable or higher rates than other racial groups, Asian Americans surgeons are significantly underrepresented in senior academic positions, societal leadership roles, and decision-making bodies.

A national analysis of orthopaedic faculty demographics found that although Asian Americans made up 11.6% of faculty, they were far less likely to hold full professorships, department chair positions, or high-impact academic roles [32]. For example, in a study of 186 academic shoulder and elbow surgeons, Asian Americans accounted for 14.5% of faculty but only 21.7% held professor titles—compared to 44.0% of their White counterparts [30].

These discrepancies cut across subspecialties. A survey of 94 adult reconstruction fellowship directors found that just 12.9% were Asian American, and only one (1%) was a woman [30]. This disparity is more pronounced at the national level: from 1990 to 2022, only 2% of the 78 presidents elected to the Hip Society, Knee Society, and American Association of Hip and Knee Surgeons (AAHKS) were Asian, and 3% were Hispanic [32].

Academic influence is similarly unequal. According to a study of racial and gender diversity in orthopaedic academic leadership, Asian Americans accounted for just 12% of Board of Directors seats, 16% of editorial board duties, and 12% of NIH orthopaedic research funding [34]. This represents a further step down from Asian Americans’ standing in the residency pipeline, emphasizing their persistent exclusion from high-level leadership and research possibilities.

Professional membership data further supports this attrition trend. From 2012 to 2018, Asian American membership in the American Academy of Orthopaedic Surgeons (AAOS) rose only slightly from 5.5% to 6.7% [24]. Even among Asian female surgeons, representation improved only modestly from 6.3% to 9.0%—still far below expected levels given their growing presence in training programs.

This persistent gap between early representation and later leadership reflects what scholars have termed the “bamboo ceiling,” which is a phenomenon describing invisible barriers that prevent Asian Americans from rising into top leadership positions despite strong qualifications and abundant representation in entry-level roles. These findings exemplify this “bamboo ceiling” idea as they show how Asian Americans may be hindered from advancing beyond technical or support roles into positions of leadership and visibility within the field. While well represented at the entry points of medical training, Asian American orthopaedic surgeons are disproportionately absent from the ranks of professors, program directors, society presidents, and grant-funded researchers. This disparity suggests not only a leadership gap but also a deeper structural exclusion from the pathways that shape the profession’s future.

### 3.7. A Leadership Gap That Undermines People-Centred Systems

This lack of representation in leadership not only limits professional advancement but also curtails the inclusion of Asian Americans’ perspectives in shaping clinical guidelines, institutional policies, and research priorities. Leadership diversity is a crucial component of health equity—ensuring that diverse patient populations are represented not only in care delivery but also in the systems that govern clinical decision-making. When Asian American physicians are absent from these roles, the health system risks overlooking nuanced cultural and linguistic needs, perpetuating disparities in care access, communication, and outcomes for Asian Americans and other underserved populations.

### 3.8. Intersectionality of Gender and Race in Orthopaedic Surgery

While this review aimed to evaluate intersectional disparities affecting AAPI women, our analysis revealed a significant limitation in the primary literature: most studies failed to report gender-specific data for AAPI subgroups. For instance, although the Pediatric Orthopaedic Society of North America (POSNA) demonstrated increased AAPI representation over time, the gender distribution within AAPI leadership was not disclosed. Similarly, national-level academic data sets reported aggregate AAPI figures without stratifying by gender, precluding a more granular understanding of compounded inequities faced by AAPI women. This lack of disaggregated data represents not just a methodological gap but a structural oversight that perpetuates the invisibility of intersectional identities in orthopaedic research and policy discussions [33].

Subspecialty distribution characteristics further revealed an even greater degree of segregation, with Asian surgeons over-represented in adult reconstruction and spine fellowships overall, while Asian women remained under-represented in those fields [28]. At the national faculty level, Asian Americans made up 11.6% of orthopaedic faculty across ACGME-accredited academic programmes [32]. However, there was no report on gender-specific data, making it difficult to understand the dual form of discrimination. Female representation in orthopaedics as a whole was lower than in other surgical fields, and there was slow progress for Asian women.

## 4. Discussion

The present study reveals a consistent pattern of Asian American underrepresentation in orthopaedic leadership positions despite modest training programme representation. The pooled analysis showing substantially reduced probability of Asian American presence at academic senior roles (RD 0.19, 95% CI [0.12, 0.28]) indicates entrenched structural impediments beyond entry to the profession [20,22,30,32,34,36]. This “bamboo ceiling” phenomenon reflects more pervasive patterns of academic medicine, wherein Asian Americans face out-of-proportion hurdles for progression while being well-represented in trainee pipelines.

The statistical heterogeneity suggests significant variability in Asian American underrepresentation across different academic institutions, subspecialties, and career stages. While the overall effect remains statistically significant (*p* < 0.00001), indicating a consistent pattern of underrepresentation, the magnitude of this disparity likely fluctuates considerably across settings. Such heterogeneity may reflect institutional differences in promotion practices, varying regional demographics, subspecialty-specific cultural factors, and differences in data collection methodologies across studies.

Asian American stereotypes label Asian Americans as technical but not leader-like, sustaining exclusion from mentorship opportunities critical to career progression [37]. Evidence of Asian American clustering in adult reconstruction and spine subspecialties indicates potential steering toward technically demanding but hidden leader tracks [28]. While these fields are intellectually rigorous as well as clinically vital, generally their leadership structures may be less centralized or accessible, which may limit a broader visibility within the academic landscape of orthopaedic. In addition, the large gap between Asian Americans who are tenured (43.00%) and non-Asians (48.65%) indicates system-level evaluation disparities that accumulate throughout career stages.

The underrepresentation of Asian American women in orthopaedic surgery highlights gender as an additional barrier to career advancement [24]. The predominance of Asian American surgeons among specific subspecialties also indicates possible exclusion from networks that enable progress to senior roles, specifically sports medicine, where representation remains disproportionately low.

Furthermore, lacking inclusion in academic and research decision-making structures may compound leadership disparities. For example, Asian Americans were completely absent from U.S.-based knee surgery guideline authorship despite being a significant demographic within orthopaedic practice [26]. Although Asian American researchers represented a sizable portion of NIH orthopaedic research grant recipients, they were still less likely to occupy influential positions on editorial boards or organizational leadership panels [34]. These findings indicate that while representation may exist at technical or contributor levels, it does not equate to authority in guiding orthopaedic discourse or policy.

In parallel, patient-level patterns of invisibility have also been reported. One study found that Asian American patients underwent total knee arthroplasty (TKA) at significantly lower rates than White patients, despite similar clinical need, and after adjusting for socioeconomic status [35]. Although patient outcomes are not the primary focus of this review, such findings reflect broader systemic marginalization that may be mirrored in the profession itself. Likewise, Asian patients undergoing ACL reconstruction were found to experience more barriers to care than White patients, despite reporting fewer socioeconomic challenges than Black and Hispanic groups [24]. This point may reflect a downstream effect of the broader lack of Asian American leadership in the field, where there is an absence of culturally aligned decision-makers to contribute to gaps in care delivery and advocacy. Together, these trends emphasize the need for leadership structures that are more reflective of and responsive to the communities they serve.

### 4.1. Study Strengths

This longitudinal analysis enables robust trend identification that distinguishes between temporary fluctuations and persistent structural patterns. In addition, the inclusion of intersectional data examining both race and gender dynamics reveals the compounded barriers facing Asian American women. Moreover, this study employed an analytical framework examining influence measures, including grant funding allocation, publication impact, and decision-making authority. In addition, by triangulating quantitative findings with contextual institutional factors, this study establishes causal links between systemic practices and career outcomes, providing actionable insights for evidence-based interventions rather than merely documenting disparities.

### 4.2. Study Limitations

Most included studies used surname analysis or self-report race and ethnicity, and thus are subject to classification errors and inter-study inconsistencies. Furthermore, grouping various Asian subgroups masks possible distinctions within and between East, South, and Southeast Asian groups. Moreover, limited qualitative data do not allow exploring lived experiences and particular obstacles to progression for Asian American orthopaedic surgeons.

Although a pooled meta-analysis was initially considered, the high heterogeneity (I^2^ = 98%) and variability in outcome definitions limited interpretability and risked oversimplifying complex structural disparities. We therefore opted against a pooled statistical estimate and instead emphasized a structured thematic synthesis across the literature—consistent with systematic review methodology when meta-analysis is not appropriate.

### 4.3. Study Implications

Institutions should adopt specialized mentorship initiatives targeting career advancement obstacles for Asian American surgeons, prioritizing leadership development rather than technical expertise. The large representation gap between the entry and senior levels emphasizes the need for interventions that target career advancement channels rather than mere recruitment.

Promotion and tenure panels should use bias reduction measures such as systematic assessment frameworks and diverse committees to counter evidence-based disparities between representation and advancement. In addition, specialized programs for Asian American orthopaedic surgeons aimed at developing their leadership abilities would address documented disparities, especially among women who encounter intersecting obstacles.

Analysis of pathways to successful career progression is essential to provide useful insights to inform the creation of focused interventions. In addition, follow-up cohort studies monitoring career pathways longitudinally would further enhance knowledge of particular career progression barriers.

## 5. Conclusions

This study underscores that the absence of Asian American surgeons from leadership roles in orthopaedic surgery is not a passive outcome of meritocracy but a reflection of structural inequities that affect the broader health system’s ability to serve diverse populations. Despite strong entry into the profession, Asian Americans are excluded from influential positions that shape educational, clinical, and policy decisions—resulting in a leadership gap that perpetuates systemic invisibility.

Beyond addressing disparities in representation, improving Asian American inclusion in leadership is vital for advancing health equity. Leaders from diverse backgrounds are more likely to advocate for inclusive practices, cultural competency, and equitable policy reform. This is especially important for addressing the unmet needs of Asian American communities, who remain underrepresented both as healthcare providers and as priority populations in orthopaedic care.

To create truly people-centred systems, institutions must prioritize equitable leadership development pipelines, ensure transparency in promotion criteria, and create inclusive environments that support career advancement for marginalized groups. By investing in Asian American leadership, orthopaedic surgery can take a critical step toward aligning its workforce with the communities it serves and toward building a healthcare system that is representative, responsive, and just. Finally, the widespread absence of gender-stratified data for AAPI populations across the included studies underscores an urgent need for future research to prioritize intersectional frameworks. Without such data, the compounded barriers faced by AAPI women remain unexamined and unaddressed, limiting the field’s capacity for truly inclusive leadership development.

## Figures and Tables

**Table 1:** Newcastle–Ottawa Scale (NOS) assessment results.

Author	Selection		Comparability	Outcome
	Representativeness of Cohort	Ascertainment of Exposure	Comparability of Cohorts	Assessment of Outcome
Chen et al. (2020) [21]	*	*	*	*
Cummings et al. (2023) [22]	*	*	*	*
Day et al. (2010) [23]	*	*	*	*
Lum et al. (2024) [24]	*	*	*	*
Mariner Gonzalez et al. (2024) [25]	*	*	*	*
Okike et al. (2011) [26]	*	*	*	*
Onuoha et al. (2022) [27]	*	*	*	*
Poon et al. (2018) [28]	*	*	*	*
Poon et al. (2019) [29]	*	*	*	*
Schiller et al. (2020) [30]	*	*	*	*
Shah et al. (2020) [31]	*	*	*	*
Silvestre et al. (2024) [32]	*	*	*	*
Singleton et al. (2021) [33]	*	*	*	*
Trenchfield et al. (2023) [7]	*	*	*	*
Vij et al. (2022) [34]	*	*	*	*
Wang et al. (2023) [35]	*	*	*	*

An asterisk (*) indicates that the study met the criterion and was awarded one point according to the Newcastle–Ottawa Scale (NOS). Empty cells indicate the criterion was not met or was not reported in the original study.

**Table 2:** Study Characteristics.

Study	Country	Study Design	Sample Size	Demographic Breakdown	Main Objective	Conclusions
Chen et al. (2020) [21]	USA	Retrospective analysis	186 academic shoulder and elbow surgeons	White: 83.9% (156/186) Asian: 14.5% (27/186)	Analyse racial and gender diversity among US academic shoulder and elbow surgeons.	White surgeons had significantly longer time in practice (mean 18.8 vs. 12.6 years, *p* < 0.01) and a higher likelihood of holding a professor rank (44.0% vs. 21.7%, *p* = 0.04) compared to nonwhite surgeons
Cummings et al. (2023) [22]	USA	Retrospective analysis	119 orthopaedic surgery residents and fellows	White 62.2% (*n* = 74), Multiracial/Other 14.3% (*n* = 17), Asian 9.2% (*n* = 11), Black 8.4% (*n* = 10), Hispanic 5.0% (*n* = 6), Native American 0.8% (*n* = 1)	To quantify diversity in race, ethnicity, gender, and sexual orientation among orthopaedic residents/fellows	Discrimination based on race, ethnicity, and gender remains a major barrier in orthopeadic training. Mentorship, especially from diverse faculty
Day et al. (2010) [23]	USA	Retrospective analysis	Medical students: 15,810 Orthopaedic residents: 3210 (2006), 3210 (2007)	White: Asian-American: African American	Compare diversity (race, ethnicity, sex) in orthopaedics versus other surgical/nonsurgical specialties at residency and faculty levels	Minority applicants submitted fewer applications than White/Asian applicants (39.6–39.8 vs. 47.3–51.7)
Lum et al. (2024) [24]	USA	Retrospective analysis	30,000 AAOS members	White: 88.5% → 84.7% African American: 1.6% → 1.8% Hispanic/LatinX: 1.8% → 2.2% Asian: 5.5% → 6.7% (Asian females: 6.3% → 9.0%) Native American: 0.3%	Analyse geographic/demographic trends in orthopaedic workforce (2012–2018)	Overall surgeon density increased (0.44 ± 0.74/100,000; *p* < 0.001) Largest density declines: DC (−3.21), Wyoming (−1.5), North Dakota (−0.87)
Okike et al. (2011) [26]	USA	Retrospective analysis	Orthopaedic residents: 1758 (1968) to 3303 (2008) annually	Asian: 11.7% African American: 4.0% Hispanic: 3.8% American Indian/Alaskan Native: 0.4% Native Hawaiian/Pacific Islander: 0.3%	Quantify minority representation trends in orthopaedic residencies vs. other specialties.	Orthopaedics remained least diverse specialty (*p* < 0.001) despite gains. Asian representation grew fastest but remained below other specialties
Onuoha et al. (2022) [27]	USA	Retrospective analysis	Orthopeadic applicants/residents compared across 8 specialties	Asian: 11.7%→14.7% *p* = 0.038 Hispanic: 5.1%→3.8%, *p* = 0.375	Evaluate diversity changes in orthopeadic applicants/residents vs. other specialties.	Orthopeadics had lowest minority (28.7%) and female (15.4%) resident representation in 2019 (*p* < 0.001) Significant gap between minority applicants (35.8%) and residents (28.7%) suggests selection bias
Poon et al. (2018) [28]	USA	Retrospective analysis	3722 orthopeadic fellows	White: 2551 (68.5%) Asian: 648 (17.4%) Hispanic: 175 (4.7%) Black: 161 (4.3%)	Characterise diversity trends in orthopaedic fellowships.	No increase in racial/ethnic minority representation over time. Subspecialty preferences: Asian: Adult reconstruction and spine White: Sports medicine, hand surgery, trauma
Poon et al. (2019) [29]	USA	Retrospective analysis	all ACGME-accredited orthopaedic residencies 2006–2015	Asian: 12.5% African American: 4.2% Hispanic: 4.5% (*p* = 0.0003 increase) American Indian/Alaskan Native: 0.36% Native Hawaiian/Pacific Islander: 0.19%. White: 74.5%	Analyse diversity trends in orthopaedic vs. other surgical residencies	Orthopaedics had: Lowest female representation (14.4% in 2015)
Schiller et al. (2020) [30]	USA	Retrospective analysis	94 Orthopaedic Surgery Adult Reconstruction Fellowships Directors (FDs)	80.65% Caucasian (*n* = 76); 12.90% Asian American (*n* = 12); 5.38% Hispanic/Latino (*n* = 4); 1.08% African American (*n* = 1)	To evaluate shared characteristics among current adult reconstruction FDs	FDs are predominantly male and Caucasian, with high research productivity (H-index)
Shah et al. (2020) [31]	USA	Retrospective analysis	1997: 1562 orthopaedic faculty 2017: 3783 orthopaedic faculty	White: 78.5% Asian: 11.6%	Evaluate URM and female representation trends in orthopaedic faculty vs. other specialties.	Orthopaedics had: Lowest URM representation (6.1%)
Silvestre et al. (2024) [32]	USA	Retrospective analysis	97 appointments of 78 unique Hip and Knee Arthroplasty presidents	95% Caucasian, 2% Asian, 3% Hispanic	To compare characteristics of presidents in the Hip Society, Knee Society, and AAHKS from 1990–2022	Presidents have high academic output and similar demographics
Singleton et al. (2021) [33]	USA	Retrospective analysis	2010; 608 Orthopaedic Surgical Society Active Members 2020: 818 Orthopaedic Surgical Society Active Members	Caucasian: 84.0% Asian: 11.2% Hispanic/Latin/South American (HLSA): 2.9% African American: 1.8%	Analyse sex/racial diversity trends in POSNA membership and leadership.	Increased diversity in all categories (2010–2020): Female members: +9.1 percentage points Asian members: +3.8 points
Trenchfield et al. (2023) [7]	USA	Retrospective analysis	all orthopeadic spine fellowship trainees from 2007–2021	White (28–66%) Asian (9–28%) Black (3–16%) Hispanic (0–10%)	To analyse trends in racial, ethnic, and gender diversity among orthopeadic spine surgery fellowship trainees.	Orthopaedic spine fellowships have not achieved significant progress in diversifying by race or gender. White males dominate, with little representation from females and underrepresented minorities.
Vij et al. (2022) [34]	USA	Retrospective analysis	Board of Directors: 307, Editorial Boards: 376, NIH Grant Recipients: 182 (Academic Orthopaedic Surgery)	Board of Directors: 72% Caucasian, 12% Asian, 1% Hispanic/Latino, 9% African American, 6% Other. Editorial Boards: 77% Caucasian, 16% Asian, 4% Hispanic/Latino, 2% African American, 1% Other	To analyse racial/ethnic and sex diversity within academic orthopaedic surgery	Academic orthopaedic surgery leadership and research bodies show a predominance of Caucasian and male individuals. Women and underrepresented minorities are significantly fewer across all academic domains analysed.
Wang et al. (2023) [35]	USA	Retrospective analysis	all individuals entering U.S. Orthopaedic surgical residencies from 2001 to 2020	Asian, Black or African American, Hispanic/Latino/Spanish Origin, NHOPI, White,	To examine trends in sex and racial representation among entering orthopaedic surgery residents in the U.S	Orthopaedic surgery improved in sex diversity but racial diversity saw less progress

## Data Availability

Data sharing is not applicable. No new data were created or analyzed in this study.
